# 1-year follow-up of the mental health and stress factors in asylum-seeking children and adolescents resettled in Germany

**DOI:** 10.1186/s12889-019-7263-6

**Published:** 2019-07-08

**Authors:** Lauritz Rudolf Floribert Müller, Katharina Gossmann, Franziska Hartmann, Karl Phillipp Büter, Rita Rosner, Johanna Unterhitzenberger

**Affiliations:** Department of Psychology, Catholic University of Eichstätt-Ingolstadt, Ostenstraße 25, 85072 Eichstätt, Germany

**Keywords:** Asylum-seeking, Children and adolescents, Unaccompanied, Mental health, Trauma, Longitudinal, Post-traumatic stress disorder, Resettlement, Minor refugees

## Abstract

**Background:**

Asylum-seeking children and adolescents (ASCs) who have resettled in Western countries show elevated rates of psychological distress, including Posttraumatic Stress Symptoms (PTSS), depression, and anxiety. Most longitudinal data suggest a relatively stable course of symptoms during the first years in exile. However, no longitudinal examination of the mental health of ASCs, who resettled in Europe in the wake of the 2015–17 European migrant crisis, has been conducted so far.

**Methods:**

A prospective cohort study looked at 98 ASCs who resettled in southern Germany throughout 2015–17. They mainly came from Afghanistan, Syria, Eritrea, and Iraq. Baseline assessments were undertaken 22 months, on average, after resettlement, and follow-up assessments 1 year thereafter. Seventy-two ASCs could be secured for the follow-up. The measures included self-report questionnaires screening for PTSS, depression, anxiety, externalizing behavior, and post-migration factors that were administered in an interview-like setting. Results were analyzed using hierarchical multiple regression analysis.

**Results:**

Participating ASCs reported on average eight potentially traumatic experiences and high levels of psychological distress at baseline that had significantly declined at follow-up. At follow-up, rates of clinically significant symptoms ranged from 9.7% (externalizing behavior) to 37.5% (PTSS). There was considerable individual variation in symptom change resulting in multiple mental health trajectories. ASCs whose asylum applications had been rejected presented significantly more symptoms than ASCs whose asylum applications had been accepted between assessments. Baseline psychopathology and asylum status predicted follow-up symptom severity.

**Conclusions:**

In contrast to earlier studies, the symptom severity in this sample of ASCs in Germany ameliorated between assessments. Decisions on the asylum applications of ASCs are thought to contribute to the course of symptoms. Since levels of psychological distress were still high, dissemination and implementation of appropriate treatments for ASCs is crucial.

**Electronic supplementary material:**

The online version of this article (10.1186/s12889-019-7263-6) contains supplementary material, which is available to authorized users.

## Background

A constantly increasing number of people worldwide are fleeing their homes on account of ongoing international armed conflicts, political oppression or persecution, and environmental changes [1, 2]. While most refugees resettle in their home or in neighboring countries [3] there has also been a substantial increase in asylum applications throughout Europe in recent years, peaking in 2015 when roughly 1.26 million people were registered as first-time asylum applicants [4]. It is estimated that half of the refugees and asylum-seekers worldwide [3] and nearly a third in Europe [4] are minors, hereinafter referred to as asylum-seeking children and adolescents (ASCs).

There is a growing body of research dedicated to the investigation of the mental health of ASCs who have resettled in Western countries. So far, research on the psychological distress levels of ASCs has clearly identified an elevated psychopathology. Posttraumatic stress symptoms (PTSS) following the frequent experience of potentially traumatic events are the most common mental health problem [5]. In addition, ASCs present additional serious mental health problems including depression, anxiety, and externalizing behavior [6–8]. While numerous studies support the findings of increased levels of psychological distress among ASCs on a cross-sectional level [5, 9], very few longitudinal investigations have been conducted so far. In essence, two inverse courses of the mental health status of ASCs within the process of post-resettlement seem feasible. Whilst symptom levels may improve thanks to the absence of migration-related adversities (e.g. war atrocities, deprivation) and the experience of safety in the host country, it could also be argued that post-migration stressors may contribute to perpetuating or even worsening the mental health status of ASCs. In addition, PTSS have mainly been shown to be mental health problems with a risk of chronification [10]. Hence, they may not simply diminish once the migration-related adversities disappear.

So far, the scarce number of longitudinal studies have yielded mixed results. Most studies that examine ASCs during their first years in exile [11–15] report no or little decline in symptoms over time, thereby supporting the latter courses of symptoms. In contrast, some studies that tracked their participants over the course of six [16] to eight and nine [17] years after resettlement, revealed some to considerable mitigation of PTSS and depression. However, for the first study [16], levels of psychological distress were shown to be chronic in nature later on [18] with rates of clinically significant PTSS of 35% 12 years after the ASCs first arrived in the host country. Moreover, in both cases, the rates of clinically relevant symptoms, including PTSS and depression, exceeded the levels of psychological problems in community samples reported elsewhere [19, 20]. Overall, this illustrates the stable nature of increased levels of psychological symptoms in ASCs in terms of a variety of mental health problems.

Nevertheless, several studies have pointed out the substantial variation in symptom levels in longitudinal studies which results in the various mental health trajectories reported in these studies [13, 17, 21, 22]. For instance, in a two-year follow-up study on unaccompanied ASCs in Norway, 40.4% of those with clinically significant PTSS at baseline decreased to below cut-off at follow-up, yet, at the same time, 47.8% of those with no clinically significant PTSS at baseline increased to above cut-off at follow-up [13]. Late-onset posttraumatic stress disorder (PTSD) has also been described by Smid and colleagues [23] where 18% developed clinically significant PTSS after a preceding period of unremarkable symptoms. Montgomery [17] attempted to categorize the course of symptoms among ASCs. This resulted in four distinct trajectories: (a) unproblematic at both assessments (hereinafter referred to as *unremarkable*), (b) problematic at first assessment only (hereinafter referred to as *adapted*), (c) problematic at follow-up only (hereinafter referred to as *reacting*), (d) problematic at both assessments (hereinafter referred to as *persisting*).

On a cross-sectional level, exposure to pre-migration violence and trauma, female sex, and being unaccompanied have repeatedly been found to be the major risk factors for poorer mental health status in ASCs [24]. Moreover, further factors related to the post-migration period (hereinafter referred to as *post-migration factors*) have been shown to be associated with symptom levels in ASCs but they have very rarely been investigated in longitudinal studies [21]. Consequently, taking post-migration factors into account might contribute to a better understanding of the course of symptoms in ASCs after resettlement.

To the authors’ knowledge, this is the first longitudinal study on the mental health of ASCs to be conducted in Germany and the first ever to draw on a sample of ASCs who resettled in Europe in the wake of what is known as the 2015–17 migrant crisis. During that time, Germany became Europe’s largest host country for asylum-seekers, including ASCs [4]. Cross-sectional research efforts indicated high levels of psychological distress in this subgroup [25, 26]. Moreover, in line with previous studies, our cross-sectional assessment of ASCs in southern Germany showed elevated levels of PTSS (56.1%), depression (33.7%), and anxiety (38.8%) [27]. In this study, we aimed to examine the longitudinal course of mental health symptoms 12 months after the initial investigation. Specifically, in line with earlier research, we (1) hypothesized that symptom levels would remain unchanged over time. We furthermore aimed to (2) examine trajectories of symptom courses, and to (3) identify in an exploratory manner individual (e.g. age), trauma-related and clinical (e.g. number of traumatic experiences), and post-migration (e.g. approval/refusal of asylum) factors that might predict the symptom levels of ASCs at follow-up.

## Methods

We used the STROBE statement [28] as a guideline for reporting cohort studies when drafting the manuscript of this study.

### Procedure

**Fig. 1:**
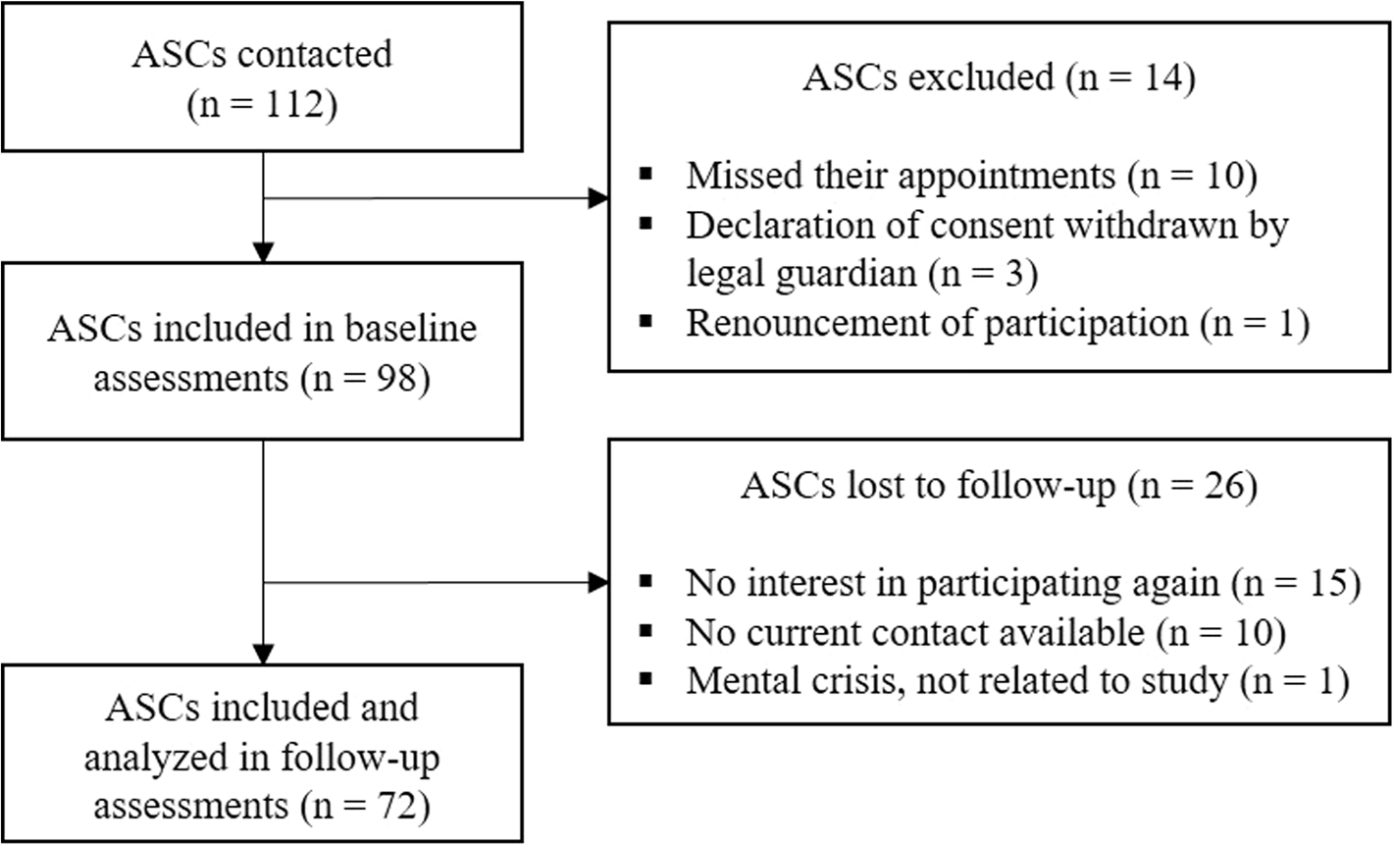
Flow diagram of the participating ASCs

This study is a prospective cohort study on the mental health and stress factors of ASCs, carried out between 2017 and 2018. In 2017, we contacted ASC facilities, refugee reception centers, and volunteers working in the field throughout Bavaria, Germany in order to promote this study among their clients. The original inclusion criteria were: an asylum-seeking minor residing in Germany for at least 3 months and written informed consent from both participants and their legal guardians. Power analysis was undertaken at baseline originally using G*Power [29] in order to identify differences between accompanied and unaccompanied ASCs, and with a moderate effect size of Cohen’s *d* = .5, a significance level of *α* = .05, a test power of 1-*β* = .8, and an allocation ratio of 1 to 2, resulting in a required sample size of *N* = 114. We had therefore recruited *N* = 112 ASCs for baseline assessments, 14 of whom dropped out before completing the assessments. This resulted in an original sample of *N* = 98 ASCs at baseline (T1) [27]. We calculated no additional power analysis prior to the follow-up, but rather contacted all youths who had given their consent to be contacted again in 2018 and arranged appointments with those who wished to participate in the follow-up assessments (T2) which took place on average 12 months (*M* = 11.8, *SD* = .58) after baseline. Altogether, *N* = 72 ASCs were available for assessment at follow-up (73.5% of the original sample). Of those lost to follow-up assessment, 15 did not want to participate again, 10 could not be contacted or located anymore, and one adolescent did not want to participate due to an urgent mental crisis that was not related to participation in the study. Figure 1 gives the participant flow.

All participants, as well as their parents or legal guardians, had given their informed consent prior to study participation at baseline but were asked to do so again at follow-up. After contacting and setting up appointments, all measures were administered in an interview-like face-to-face setting in a private room in the adolescents’ residences. All interviewers were bachelor-level psychologists and were in their master’s program by the time of the respective assessments. They had been trained and were being supervised by experienced and licensed clinicians in the context of trauma and refugees (JU, RR) at all times. If the participants did not have sufficient language proficiency, interpreters were on hand but were not needed. The interviews began by clarifying the study aims, informing the participants about the obligation of the professionals involved to uphold confidentiality, and stressing the voluntary nature of participation. All participants were given information about psychosocial services in the vicinity. The researchers provided immediate psychological support in the event of any distress caused by the assessment among the participants. However, no emergencies occurred during the study. Participants were given a 20-euro voucher as an incentive.

The study was approved by the university’s ethics board in December 2016 (ethics approval number: 2016/23).

### Participants

Seventy-two ASCs were analyzed at follow-up. The majority were boys (*n* = 65, 90.3%), of Islamic faith (*n* = 59, 84.3%), unaccompanied (*n* = 47, 65.28%), and attending school in Germany (*n* = 64, 88.9%). Half of the participants came from Afghanistan (*n* = 36, 50%), further major countries of origin were Eritrea (*n* = 9, 12.5%), Syria (*n* = 8, 11.1%), and Iraq (*n* = 7, 9.7%). Most participants were living in either semi-care (*n* = 27, 37.5%) or full-care units of the Child and Youth Welfare System (*n* = 18, 25%), in an apartment with some family member (*n* = 18, 25%), or in an apartment on their own (*n* = 8, 11.1%). The asylum applications of most participants had been accepted (*n* = 51, 71.8%), 25 of which had been accepted between baseline and follow-up assessment (34.7%). Another 16 participants’ applications had been declined (22.5%), and another five applications were pending (6.9%). The mean age was 17 (*M* = 17.32, *SD* = 1.93) and participants had been living in Germany for almost 3 years (*M* = 2.87, *SD* = 0.74). Thirteen participants (18.1%) had undergone some sort of psychosocial intervention since their resettlement in Germany but did not differ from those with no history of treatment in terms of psychopathology. See Additional file 1: Table S1 for more information on participants’ sociodemographic characteristics.

ASCs who had completed the baseline assessment but had dropped out by follow-up did not differ from participants included in the follow-up with regard to socio-demographic data and mental health outcomes.

### Measures

The same measures were used at baseline and at follow-up. The socio-demographic background was queried via the self-report. Besides common demographic data, we also asked about any changes in placement, asylum status, or psychosocial treatment that might have occurred between baseline and follow-up. Moreover, the following measures were administered in the German versions.

The *Child and Adolescent Trauma Screen* (CATS; [30]) consists of two parts. First, participants were shown a list of 15 potentially traumatic events and were asked to indicate which of them they had ever experienced (CATS trauma list). We added another four items related to the experience of flight and resettlement (deprivation for several days, dangerous journey (e.g. on a crowded boat), imprisonment and abduction, and the committing of acts of violence (voluntarily or involuntarily)). Second, respondents rated the frequency of PTSS over the course of the previous 2 weeks (CATS symptom scale) on a four-point Likert scale. The CATS symptom scale ranges from 0 to 60, and its authors have suggested a cut-off value of 21 as an indicator of clinically significant levels of PTSS. Lastly, respondents rated the impairment caused by PTSS over the course of the previous 2 weeks by means of five dichotomous items. The CATS is based on DSM-5 criteria [31]. It has shown good psychometric properties [30] and has been used among ASC populations [32]. In this study, the inter-item reliability of the CATS symptom scale was good (20 items; α = .83).

The *Hopkins Symptom Checklist-37 for Adolescents* (HSCL-37A; [33]) is a self-report questionnaire that screens for internalizing symptoms such as anxiety and depression, and externalizing behavior. Respondents rated the frequency of symptoms over the course of the previous month on a 4-point Likert scale. The subscales are composed as follows: total score (range 37–148, cut-off 63), depression (range 15–60, cut-off 33), anxiety (range 10–40, cut-off 20), and externalizing cluster (range 12–48, cut-off 19). The depression scale and the anxiety scale add up to the internalizing cluster (range 25–100, cut-off 54). The HSCL-37A has been widely used among diverse populations and shows good psychometric properties [33]. In this study, inter-item reliability for the constituent scales was as follows: total score (37 items, α = .89), depression (15 items, α = .86), anxiety (10 items, α = .78), externalizing cluster (12 items, α = .54), and internalizing cluster (25 items, α = .90).

The *Everyday Resources and Stressors Scale* (ERSS, Müller & Büter, unpublished scale) is a 20-item self-report questionnaire designed to screen for the following post-migration factors: experience of discrimination, social support within the family, social support in the host country, language proficiency, and everyday resources (all ranging from 1 to 4). One subscale has been derived from the Everyday Discrimination Scale [34]. The other subscales were composed by the construction of items concerning relevant post-migration factors identified through consensual literature research [24, 35]. The ERSS is yet to be validated but it was successfully used at baseline with α ranging from .71 to .77 [27]. In this study, the inter-item reliability of discrimination (4 items, α = .45) and the social support in the host country (5 items, α = .33) subscales were psychometrically unsatisfactory and were, therefore, excluded from the analysis. The inter-item reliability of the other scales was as follows: social support within the family (3 items, α = .81), language proficiency (3 items, α = .74), and everyday resources (5 items, α = .68).

### Statistical analyses

Data were analyzed using SPSS statistics, version 25. As regards missing data, only nine single item values were missing (0.2%) across all participants and mental health outcome measures. The most conservative values were subsequently interpolated into these missing values. First, descriptive statistics were run to analyze the study sample with regard to socio-demographic data. Comparisons between groups (e.g. non-participants and participants) were conducted using *χ*^2^-statistics and independent *t*-tests. Second, Pearson’s correlations were carried out between all continuous variables at both time points. Third, paired-sample *t*-tests were used to examine differences in numbers of traumatic experiences and mental health outcomes between baseline and follow-up. Cohen’s *d* was calculated in order to indicate the magnitude of effects. In addition, the Reliable Change Index (RCI) was used to compute the critical difference. Fourth, a set of ANOVAs with Bonferroni-Holm post-hoc testing was conducted with ‘change in asylum status between time points’ as the fixed factor and mental health outcomes as the dependent variables. The subgroups were categorized as follows: asylum application pending or rejected at T2, asylum application accepted both at T1 and T2, and asylum application accepted between assessments. Finally, hierarchical multiple regression analysis was conducted in order to investigate predictors of mental health outcomes at follow-up (CATS symptom scale, HSCL-37A measures). Baseline variables that were significantly correlated with follow-up mental health outcomes were entered in a stepwise manner in the subsequent regression analysis with follow-up mental health outcomes as the dependent variables. We used residualized change score modelling as a standard approach to control for initial symptom levels (i.e., follow-up values with baseline values taken into account). The predictor variables that were entered in the regression analysis included socio-demographic data (e.g. age), numbers of traumatic experiences, mental health outcomes, and ERSS scales, all at baseline, as well as change in asylum status as described above. Baseline mental health outcome measures that were shown to be multicollinear to at least one further predictor variable were excluded from the analysis (according to VIF ≥ 3).

## Results

### Course of symptoms

Table 1 gives the numbers of traumatic experiences and all mental health outcomes at baseline and follow-up. At follow-up, the levels of clinically significant symptoms (i.e., number of participants above cut-off values) ranged from 9.7% (HSCL-37A externalizing cluster) to 37.5% (CATS symptom scale). There was a major change in symptom severity for all mental health outcome measures except for the externalizing cluster, *t* (71) = .35, *p* = .73. All symptom reductions within 1 year were found to be highly significant (on a significance level of *p* < .001) and of moderate effect size (range: depression, *d* = .41 to PTSS, *d* = .52). The numbers of traumatic experiences did not change from baseline to follow-up, *t* (71) = .67, *p* = .51, with an average of eight (*M* = 8.32, *SD* = 2.92) reported potentially traumatic experiences at follow-up. Symptom levels at baseline and follow-up were significantly correlated, and these correlations were moderate (range: PTSS, *r* = .41 to HSCL-37A total score, *r* = .60). Altogether, except for the HSCL-37A total score and externalizing cluster, the number of participants with clinically significant symptoms (i.e., above cut-off values) had declined significantly at follow-up.

**Table 1:** Mental health outcome measures at baseline and follow-up and their change scores between time points for 72 ASCs

	T1		T2						
	*M* (*SD*)	Subjects above cut-off *n* (%)	*M* (*SD*)	Subjects above cut-off *n* (%)	Change scores^a^ *M* (SD)				
						*r* ^*b*^	*t* (df)	*χ*^b^ (df)	*d*
CATS-TL	8.56 (2.96)		8.32 (2.92)		−.19 (2.97)	.48***	.67 (71)		.08
CATS-SS	22.17 (9.68)	41 (56.9)	17.29 (9.17)	27 (37.5)	−4.88 (10.26)	.41***	4.03*** (71)	5.46* [1]	.52
HSCL-Tot	63.83 (13.76)	23 (31.9)	58.33 (12.65)	13 (18.1)	−5.5 (11.93)	.60***	3.91*** (71)	3.70 (1)	.42
HSCL-Dep	30.1 (7.82)	28 (38.9)	26.96 (7.7)	12 (16.7)	−2.25 (4.79)	.49***	3.38*** (71)	8.86** [1]	.41
HSCL-Anx	18.57 (5.69)	23 (31.9)	16.32 (4.13)	12 (16.7)	−3.14 (7.88)	.56***	3.99*** (71)	4.57* [1]	.45
HSCL-Int	48.67 (12.61)	23 (31.9)	43.28 (11.17)	11 (15.3)	−5.39 (11.1)	.57***	4.12*** (71)	5.54* [1]	.45
HSCL-Ext	15.17 (2.68)	4 (5.6)	15.06 (2.91)	7 (9.7)	−.11 (2.68)	.54***	.35 (71)	.89 (1)	.04

^a^Negative values represent improvement on the respective scale

^b^between T1 and T2

*CATS-TL* CATS trauma list, *CATS-SS* CATS symptom scale, *HSCL-Tot* HSCL-37A total score, *HSCL-Dep* HSCL-37A depression scale, *HSCL-Anx* HSCL-37A anxiety scale, *HSCL-Int* HSCL-37A internalizing cluster, *HSCL-Ext* HSCL-37A externalizing cluster

*p < .05, **p < .01, ***p < .001

### Mental health trajectories

**Fig. 2:**
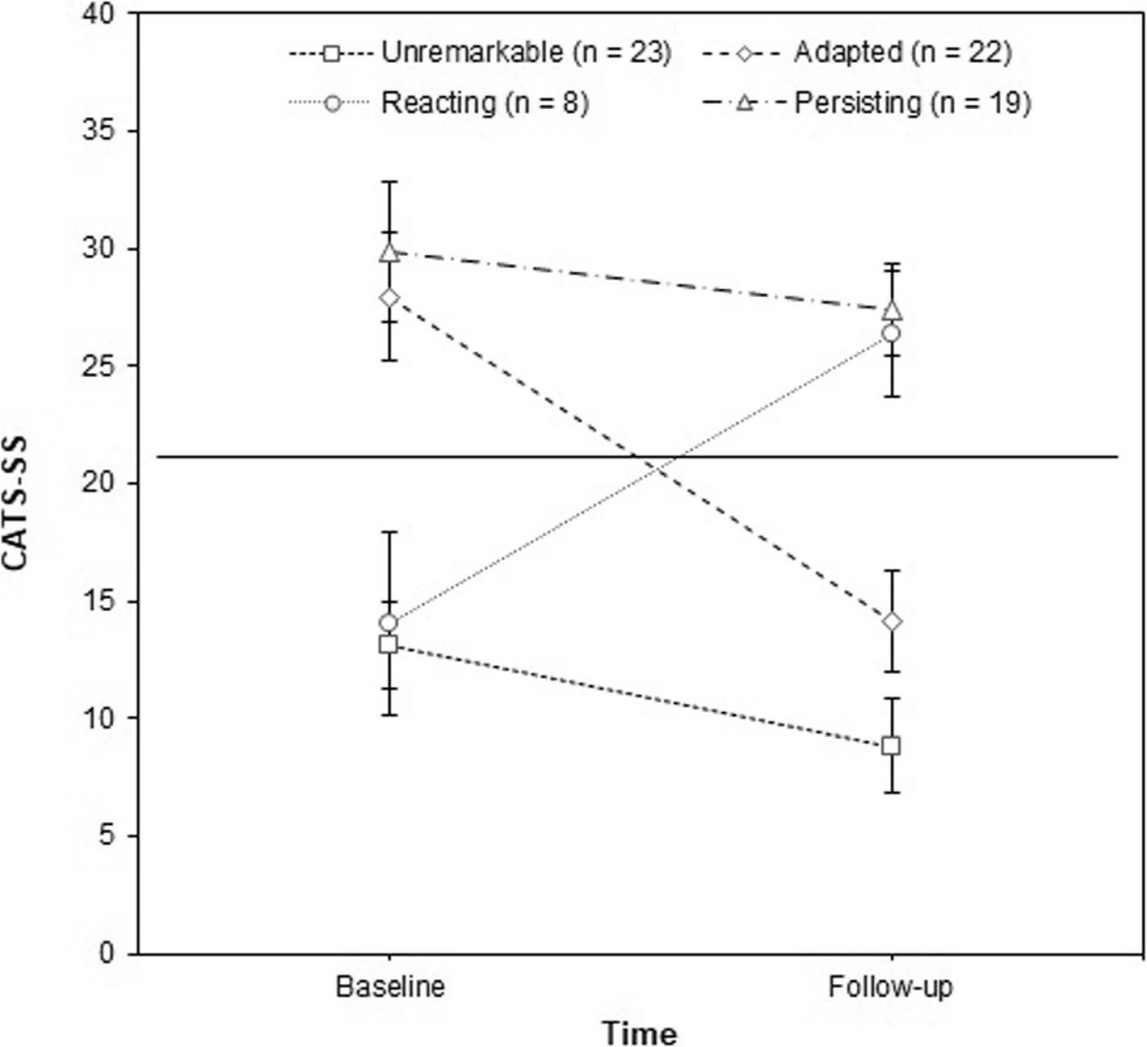
PTSS trajectories of 72 ASCs between baseline and follow-up assessments. Legend: Means and confidence intervals for PTSS trajectories of the study sample. The horizontal line represents the clinical cut-off of 21 points (CATS symptom scale)

From the descriptive angle, all mean symptom change scores showed considerable standard deviations, indicating a large variation in participants’ mental health trajectories. Table 2 gives an overview of the trajectories of all mental health outcomes over the course of 1 year. Figure 2 illustrates these transitions for the CATS symptom scale. Similar numbers of participants either showed unremarkable (*n* = 23, 31.9%) or adapted symptoms (*n* = 22, 30.6%) between assessments. Nineteen (26.4%) participants showed persisting clinically significant PTSS, and eight (11.1%) participants fell within the ‘reacting’ category. Similar trajectories emerged for the HSCL-37A. Most participants either showed unremarkable (range: depression, *n* = 39, 54.2% to externalizing cluster, *n* = 64, 88.9%) or adapted symptoms (range: externalizing cluster, *n* = 1, 1.4% to depression scale, *n* = 21, 29.2%). However, there were still large proportions of participants with persisting (range: externalizing cluster, *n* = 3, 4.2% to total score, *n* = 9, 12.5%) and reacting symptoms (range: internalizing cluster, *n* = 3, 4.2% to depression, *n* = 5, 6.9%). Moreover, as can be seen in Table 2, RCIs indicated similar proportions of change in symptom levels, with reliable improvement ranging from 19.4% (HSCL-37A anxiety scale) to 29.2% (CATS symptom scale), and reliable worsening ranging from 1.4% (HSCL-37A anxiety scale) to 9.7% (HSCL-37A depression scale).

**Table 2:** Trajectories of mental health outcome measures over the course of one year among 72 ASCs

	Unremarkable *n* (%)	Reacting *n* (%)	Adapted *n* (%)	Persisting *n* (%)	RCI	Subjects improving acc. RCI *n* (%)	Subjects worsening acc. RCI *n* (%)
CATS-SS	23 (31.9)	8 (11.1)	22 (30.6)	19 (26.4)	11.06	21 (29.2)	5 (6.9)
HSCL-Tot	45 (62.5)	4 (5.6)	14 (19.4)	9 (12.5)	13.21	16 (22.2)	4 (5.6)
HSCL-Dep	39 (54.2)	5 (6.9)	21 (29.2)	7 (9.7)	8.94	15 (20.8)	7 (9.7)
HSCL-Anx	45 (62.5)	4 (5.6)	15 (20.8)	8 (11.1)	6.5	14 (19.4)	1 (1.4)
HSCL-Int	46 (63.9)	3 (4.2)	15 (20.8)	8 (11.1)	11.59	17 (23.6)	5 (6.9)
HSCL-Ext	64 (88.9)	4 (5.6)	1 (1.4)	3 (4.2)	5.09	2 (2.8)	2 (2.8)

Unremarkable = no symptoms at T1 and T2; Reacting = no symptoms at T1, symptoms at T2; Adapted = symptoms at T1, no symptoms at T2; Persisting = symptoms at T1 and T2. RCI = critical difference according to reliable change index formula

### Group differences

Unaccompanied and accompanied ASCs did not differ significantly with respect to follow-up mental health outcomes and symptom change scores. However, there were significant main effects for change in asylum status with regard to all follow-up mental health outcome measures, except for the externalizing cluster, *F* (2, 69) = .98, *p* = .26. Bonferroni-Holm post-hoc testing revealed that participants whose applications were still pending or rejected at follow-up showed significantly more symptoms than participants whose asylum applications had been accepted between assessments. This association was found to be the largest in terms of the internalizing cluster, *t* (44) = 3.54, *p* < .01, and the magnitude of effects proved to be large (range: PTSS, *d* = .79 to internalizing cluster, *d* = 1.05). Table 3 gives the mean mental health outcomes for ASCs whose asylum applications had been accepted between assessments, ASCs whose applications had been accepted already at baseline, and ASCs whose applications were still pending or had been rejected at follow-up.

**Table 3:** Mean comparisons of mental health outcome measures at T2 between ASCs in terms of asylum application status

	(1) Asylum application accepted between assessments, *n* = 25	(2) Asylum application accepted both at T1 and T2, *n* = 26	(3) Asylum application pending or rejected at T2, *n* = 21	ANOVA main effect asylum status *F (df)*	*t* (df)^a^	
	*M* (*SD*)	*M (SD)*	*M* (*SD*)			*d* ^a^
CATS-SS	14.2 (9.68)	16.92 (9.35)	21.43 (8.52)	3.87^*^ (2, 69)	2.87^*^ (44)	.79
HSCL-Tot	54.32 (10.84)	57.85 (14.01)	63.71 (11.45)	3.4^*^ (2, 69)	2.85^*^ (44)	.84
HSCL-Dep	24.4 (5.8)	26.04 (8.33)	31.14 (7.48)	5.22^**^ (2, 69)	3.44^**^ (44)	1.02
HSCL-Anx	14.72 (3.68)	16.31 (4.07)	18.24 (4.07)	4.55^*^ (2, 69)	3.08^*^ (44)	.91
HSCL-Int	39.12 (8.97)	42.35 (11.67)	49.38 (10.71)	5.6^**^ (2, 69)	3.54^**^ (44)	1.05
HSCL-Ext	15.2 (2.53)	15.5 (3.41)	14.33 (2.63)	.98 (2, 69)	−1.14 (44)	−.34

^a^Between (1) and (3)

*CATS-SS* CATS symptom scale, *HSCL-Tot* HSCL-37A total score, *HSCL-Dep* HSCL-37A depression scale, *HSCL-Anx* HSCL-37A anxiety scale, *HSCL-Int* HSCL-37A internalizing cluster, *HSCL-Ext* HSCL-37A externalizing cluster

^*^p < .05 (adjusted level of significance according to Bonferroni-Holm*, p* < .0167)

^**^p < .01 (adjusted level of significance according to Bonferroni-Holm, *p* < .003)

### Predictors of follow-up symptom levels

Next, we investigated which factors contributed to follow-up symptom levels. Table 4 gives an overview of the results of the hierarchical regression analysis. As can be seen, baseline symptom scores were the most robust single predictors for all mental health outcomes at follow-up, except for the CATS symptom scale which was mainly predicted by HSCL-37A depression scores, *F* (1, 69) = 15.62, *p* < .001. They alone accounted for 20.7% (HSCL-37A depression scale) to 33% (HSCL-37A total score) of variance in the respective mental health outcomes at follow-up. In addition to baseline symptom scores, change in asylum status was found to predict all mental health outcome measures, except for the externalizing cluster. It was the second most robust predictor and accounted for 4.7% (HSCL-37A total score) to 8.1% (HSCL-37A internalizing cluster) of variance in follow-up symptom severity. Besides that, the only further predictor variable that contributed to variance in follow-up symptom levels was ‘time since resettlement’. It accounted for some 4% of HSCL-37A anxiety scores, Δ*R*^*2*^_*adj*_ = .043, *F* (3, 67) = 6.05, *p* < .05.

**Table 4:** Hierarchical linear regression analysis for variables predicting CATS and HSCL-37A measures at follow-up

Step	Included variable	*B* ^a^	*SE B* ^a^	Stand. β^a^	*F* change	Overall *F*	Adj. *R*^2^ change	Total adj. *R*^2^
CATS-SS								
Step 1	HSCL-Dep (T1)	.47	.13	.40^***^	15.62^***^	15.62^***^	.173	.173
Step 2	Asylum status^b^	−3.02	1.19	−.27^*^	6.41^*^	11.63^***^	.06	.233
HSCL-Tot								
Step 1	HSCL-Total (T1)	.51	.09	.55^***^	30.34^***^	30.34^***^	.33	.33
Step 2	Asylum status	−3.6	1.48	−.23^*^	8.53^*^	21.95^***^	.047	.374
HSCL-Dep								
Step 1	HSCL-Dep (T1)	.43	.1	.43^***^	19.24^***^	19.24^***^	.207	.207
Step 2	Asylum status	−2.8	.96	−.30^**^	8.5^**^	14.91^***^	.077	.284
HSCL-Anx								
Step 1	HSCL-Anx (T1)	.36	.07	.50^***^	31.01^***^	31.01^***^	.3	.3
Step 2	Asylum status	−1.42	.47	−.28^**^	8.48^**^	21.43^***^	.069	.369
Step 3	Time since resettlement	−.11	.04	−.23^*^	6.05^*^	17.36^***^	.043	.412
HSCL-Int								
Step 1	HSCL-Int (T1)	.46	.08	.52^***^	31.05^***^	31.05^***^	.3	.3
Step 2	Asylum status	−4.12	1.3	−.3^**^	10^**^	22.55^***^	.081	.381
HSCL-Ext								
Step 1	HSCL-Ext (T1)	.58	.11	.54^***^	28.63^***^	28.63^***^	.283	.283

^a^for the final model/step

^b^asylum status interval-scaled: rejected or pending at T2, accepted both at T1 and T2, accepted between assessments

*CATS-SS* CATS symptom scale, *HSCL-Tot* HSCL-37A total score, *HSCL-Dep* HSCL-37A depression scale, *HSCL-Anx* HSCL-37A anxiety scale, *HSCL-Int* HSCL-37A internalizing cluster, *HSCL-Ext* HSCL-37A externalizing cluster, *T1* baseline assessment

^*^p < .05, ^**^p < .01, ^***^p < .001

## Discussion

This study investigated the course and predictors of the most common mental health outcomes among a cohort of 72 ASCs predominantly from Afghanistan, Syria, Eritrea, and Iraq who resettled in southern Germany in the wake of the 2015–17 European migrant crisis within their first years in exile. Hence, it is the first longitudinal study on the mental health of ASCs in Germany and the first ever on ASCs who resettled during this crisis. The study demonstrated that levels of psychological distress in ASCs were high, even almost 3 years after resettlement and with two thirds of the participants having been granted asylum. At the same time, it revealed a considerable change in symptom severity with respect to PTSS, depression, and anxiety between baseline (on average 22 months after resettlement) and follow-up (1 year after baseline assessment). Participants significantly improved on all but one mental health outcome (the externalizing cluster) that were covered in the study, and the magnitude of effects was moderate. Still, a substantial number of the participants showed elevated psychopathology, with rates of clinically significant symptoms ranging from 9.7% (externalizing behavior) to 37.5% (PTSS) at follow-up, indicating a considerable need for professional support. The attrition rate from baseline to follow-up was 26.5% and was, therefore, comparable to the rates reported in studies with similar follow-up periods where attrition rates ranged from 19.4% [13] to 39.1% [12].

The results of this study are contrary to most longitudinal studies on the mental health of ASCs during the first years after resettlement that found little or no change in symptoms over time, indicating the stability and chronicity of symptoms [11–15]. Most studies that have reported an alleviation of symptoms either showed poor methodological quality (i.e., retrospective assessment of symptoms [36]), or had longer follow-up periods that tracked their participants over the course of up to 9 years [17]. Hence, this study seems to be the first prospective investigation using standardized measures to report on the improvement in the mental health status of ASCs during their first years in exile.

Given the somewhat unambiguous character of previous findings that suggest chronicity of symptoms in ASCs, the question arises as to which factors might account for the change in symptom severity in this sample of ASCs in Germany. Generally speaking, it is plausible to say that at least some symptom changes may have occurred on account of regression to the mean, a statistical phenomenon commonly described in repeated measurements designs [37]. In this context, the change in the symptoms of ASCs may reflect natural variation (i.e., random error) in repeatedly measured values rather than any real change triggered by actual symptom alleviation. Regression to the mean seems to occur primarily following particularly extreme measurements when it is more likely that subsequent measurements of the same unit will yield less extreme results [37].

However, one can also presume that real change has occurred between baseline and follow-up assessments. First, participants whose asylum applications had been accepted between the two assessments showed significantly fewer severe symptoms at follow-up on all but one mental health outcome measure (the externalizing cluster) than participants with rejected applications, and the change in asylum status significantly predicted follow-up symptom severity. Given the relatively large number of participants who had their applications accepted at follow-up (*n* = 51, 71%) as opposed to a Norwegian study on ASCs where roughly half of the participants were rejected [12], it seems likely that this disparity explains the current improved course of symptoms compared to the Norwegian sample with stable courses. In this way, the current findings add to the existing evidence suggesting an adverse effect of negative asylum decisions on the mental health of ASCs [12, 38]. It is, however, worth noting that the biggest differences in mental health outcomes were observed between those whose applications were accepted between assessments and those whose applications had been rejected at follow-up. Participants whose applications had already been accepted at baseline assessment still showed fewer severe symptoms than those with rejected applications, yet, these differences fell short of statistical significance. Hence, it might be that the positive decision on an asylum application constitutes a short-term protective factor that is superimposed by sustained psychopathology and further post-resettlement hardships that emerged at a later stage.

Apart from that, it also seems plausible that symptoms were mitigated by the absence of migration-related adversities and the experience of safety in the host country. By the time of follow-up assessments, most participants were resident in semi- or full-care units assisted by the Child and Youth Welfare System, and only few participants (*n* = 8, 11%) were living on their own. Given the detrimental effects of low-support living arrangements that have been demonstrated in different studies [12, 39], the mental health status of ASCs might have improved because of the support measures they were consistently receiving as opposed to, for instance, Jakobsen and colleagues’ study where more than 25% of the study sample were subsequently placed in centers for adults without any specialized care [12]. Since only a small proportion of the current sample had received some form of psychosocial support—and as they did not differ from those with no history of treatment— we can, moreover, assume that symptom mitigation did not occur on account of treatment effects. Quite the contrary, our results demonstrate that ASCs rarely receive the mental health care they need.

Furthermore, in line with longitudinal data from the Netherlands [11], baseline symptom scores were found to be the most robust predictors of follow-up symptom severity. With respect to PTSS severity at follow-up, baseline depression scores turned out to be the major predictor, an association that has also been repeatedly reported in longitudinal studies [21]. Still, it is a remarkable association of symptoms given that it has been reported elsewhere that late-onset PTSD might be a secondary consequence of the worsening of depressive symptoms [23].

Besides baseline psychopathology and asylum status, only one further predictor of follow-up symptom levels could be derived. Time since resettlement accounted for some variance in follow-up anxiety scores which reflects the overall symptom improvement in this study over time. However, it still has to be borne in mind that time since resettlement merely accounted for small degrees of variance in anxiety scores, and further post-migration factors did not contribute to variance in follow-up symptom scores. Quite the opposite, two scales of the ERSS were excluded from the analysis due to low internal consistency, arguably because the ERSS constitutes a new measure that is yet to be modified and validated. Therefore, post-migration factors were not found to be the major predictors of follow-up symptom severity. More standardized measures screening for post-migration factors in a concise manner are needed, and post-migration factors should be examined more rigorously in future research as they have rarely been taken into account in longitudinal investigations [21].

Despite the contrary finding on symptom mitigation, it is worth noting that this study fits in with a number of previous studies that reported considerable individual symptom variation throughout the study runtime regardless of the overall symptom course [13, 17, 22]. Symptom courses that appear to be stable at first sight then transpire to have multiple trajectories. For instance, in a Norwegian 2-year follow-up study there were equally substantial groups of participants who either decreased to below or increased to above cut-off at follow-up with regard to most mental health outcomes [13]. Similar trajectories have been described in this study, with the ‘unremarkable’- and the ‘adapted’ groups being the most prevalent. Unfortunately, no statistical analyses could be conducted as to which factors predicted the constituent trajectories because of the small subsamples. So far, Montgomery [17] has documented that the number of pre-migration traumata differentiated the ‘unremarkable’ from the ‘persisting’ type, whereas the experience of stressful life events after resettlement differentiated ‘adapted’ ASCs from those with ‘persisting’ symptoms. This is largely in line with the Norwegian study where the increase in reported stressful life events predicted worsening of PTSS [13]. Furthermore, a Dutch study has found that late-onset PTSD (i.e., the ‘reacting’ type) was associated with older age, low levels of education, and numbers of pre-migration traumata, the latter of which were, however, fully mediated by depression and anxiety scores [23]. In summary, the factors associated with the mental health trajectories of ASCs have been detected sporadically but should be further examined in order to fully understand individual transitions. Moreover, more studies that include longer follow-up periods are necessary in order to inform clinicians about the natural history of symptoms. Both approaches could enable us to better monitor associated conditions in ASCs and derive implications for tailored mental health care.

## Limitations

Certain limitations should be borne in mind when interpreting the results of this study. Even though the attrition rate from baseline to follow-up was rather low, one major drawback of the study is the small sample size. A larger sample would have allowed for more nuanced statistical analyses regarding different subgroups of the current sample. Moreover, recruiting a random sample would have resulted in enhanced representativeness because distortions on the basis of selection bias could have been obviated. In addition, checklists instead of clinical interviews were used which means that the determined rates of psychological distress merely reflect an estimation of psychopathology. Even though these measures have been used broadly in research with ASCs and a good reliability has been documented consistently in these studies, it still deserves some consideration if cultural validity of these measures can be assumed for this particular population. Inquiring into the perceptions of ASCs with regard to the cultural appropriateness of the measures could therefore constitute a worthwhile addition to fathoming their overall validity. Other areas of psychological problems could not be covered nor were other potentially relevant factors such as hope and coping styles that could constitute important sources of resilience in ASCs. Furthermore, there were only two assessments, with the baseline taking place almost 2 years after the resettlement of the ASCs. Consequently, neither initial mental health transitions following the very early stages of resettlement nor long-term symptom changes could be examined. Finally, different interviewers administered the follow-up assessments and the baseline assessments. It is not, therefore, clear whether any sort of experimenter effect might have occurred. Given these limitations, the results of this study should be generalized with caution to ASCs as a whole but may function as a starting point for further long-term research.

## Conclusions

We discussed several explanations for the significantly decreased psychological symptoms in our sample of ASCs after 1 year, including the impact of asylum status, the experience of safety and current living arrangements, and regression to the mean.

In conclusion, it is, on the one hand, somewhat promising to observe that a substantial number of the participating ASCs seems to show an improvement in symptoms, and that, even 3 years after resettlement, most ASCs were still living in specialized care units. On the other hand, the current findings do not belie the fact that, despite improvement, the levels of psychological distress among ASCs were still high and exceeded those reported in community samples [19, 20]. In addition, a shortage of mental health care was documented, underlining the need for a functional system for meeting the psychological needs of ASCs, both on a community and on an individual level. Psychosocial interventions should address the nexus between mental health problems on the one hand, and post-migration stressors and acculturative hardships on the other. As rates of PTSS, depression, and anxiety were high and have been shown to be equally prevalent in the refugee population [8], interventions should encompass addressing not only PTSS, but also depression and anxiety. Even though it is, yet, a scarce number, there are several interventions being put forward, including prevention groups [32] and individual therapy [8, 40], that have proved to be promising approaches. They should be integrated into national health care systems, and preferably embedded in stepped-care and cross-sectoral models as suggested by Horlings & Hein [41] and Fazel & Betancourt [42] in order to address the wide array of stressors encountered by ASCs.

## Additional file

Additional file 1: **Table S1.** Sociodemographic characteristics of the participating ASCs (*n* = 72) at follow-up. (DOCX 4408 kb)

## Data Availability

The datasets generated and analyzed during this study are not publicly available but are available from the corresponding author on reasonable request.

## Abbreviations

ASCs: Asylum-seeking children and adolescents
CATS: Child and Adolescent Trauma Screen
ERSS: Everyday Resources and Stressors Scale
HSCL-37A: Hopkins Symptom Checklist-37 for Adolescents
PTSD: Posttraumatic Stress Disorder
PTSS: Posttraumatic stress symptoms
RCI: Reliable Change Index
VIF: Variance inflation factor
