# Supplementary material for: 1-year follow-up of the mental health and stress factors in asylum-seeking children and adolescents resettled in Germany
Source: BMC Public Health. 2019 Jul 8;19:908. doi: 10.1186/s12889-019-7263-6 (PMC6615278; doi:10.1186/s12889-019-7263-6)
Supplement: Supplementary file 1 — Table S1. Sociodemographic characteristics of the participating ASCs (n = 72) at follow-up. (DOCX 4408 kb) [file 12889_2019_7263_MOESM1_ESM.docx]

| *Table S1*  Sociodemographic characteristics of the participating ASCs (*n* = 72) at follow-up | |
| --- | --- |
| Age in years, *M* (*SD*, range) | 17.32 (1.93, 12–20) |
| Gender, *n* (%)  male  female | 65 (90.3)  7 (9.7) |
| Country of origin, *n* (%)  Afghanistan  Syria  Eritrea  Iraq  Gambia  Somalia  Bangladesh  Ethiopia  Iran  Mali | 38 (52.8)  9 (12.5)  9 (12.5)  7 (9.7)  3 (4.2)  2 (2.8)  1 (1.4)  1 (1.4)  1 (1.4)  1 (1.4) |
| Religion, *n* (%)  Islam  others | 60 (83.3)  12 (16.7) |
| Length of stay in months, *M* (*SD*, range) | 34.47 (8.82, 12–72) |
| Asylum status, *n* (%)  accepted  at baseline  between assessments  rejected  pending | 51 (70.8)  26 (36.1)  25 (34.7)  16 (22.2)  6 (7) |
| Being unaccompanied, *n* (%) | 48 (66.7) |
| Number of moves in past year, *n* (%)  0  1  >1 | 42 (58.3)  25 (34.7)  5 (7) |

**Additional File 1**
